# Development of a controlled-environment assay to induce iron deficiency chlorosis in soybean by adjusting calcium carbonates, pH, and nodulation

**DOI:** 10.1186/s13007-022-00855-5

**Published:** 2022-03-21

**Authors:** R. Merry, M. J. Espina, A. J. Lorenz, R. M. Stupar

**Affiliations:** Department of Agronomy and Plant Genetics, University of Minnesota, Saint Paul, MN 55108 USA

**Keywords:** Iron deficiency chlorosis, Soybean, Growth chamber assay, Nodulation

## Abstract

**Background:**

Soybean iron deficiency chlorosis (IDC) is an important nutrient stress frequently found in high pH and/or soils high in calcium carbonates. To advance the understanding of IDC resistance in soybean, a rapid (21-day) controlled-environment assay was developed to investigate the effects of nodulation, pH, and calcium carbonate levels on soybean iron deficiency traits. This system was tested on four genotypes known to exhibit differences in iron efficiency, including two standard IDC check cultivars and a pair of near-isogenic lines exhibiting variation at an IDC resistance quantitative trait locus. Visual score, chlorophyll content, plant height, root dry mass, and shoot dry mass were measured to quantify iron stress.

**Results:**

Calcium carbonate levels and nodulation were found to have the greatest effects on IDC severity. Increasing calcium carbonate levels worsened IDC symptoms, while nodulation reduced symptoms in all genotypes. Higher pH levels increased iron deficiency symptoms in check genotypes ‘Corsoy 79’ and ‘Dawson’, but did not induce iron deficiency symptoms in near-isogenic lines. A significant interaction was observed between genotype, nodulation, and calcium carbonate level, indicating that a specific treatment level could discern IDC symptoms between genotypes differing in resistance to IDC.

**Conclusions:**

IDC symptoms were successfully induced in the Check Genotypes Experiment as well as the NIL Experiment, indicating the success of using this assay for inducing IDC in controlled environments. However, our results suggest that treatment levels that best differentiate genotypes for their IDC resistance may need to be determined for each experiment because of the unique way in which different genotypes display symptoms and respond to iron deficiency conditions.

## Core ideas

- An assay was developed to independently assess factors leading to IDC in soybean.
- Calcium carbonates have a greater influence on IDC symptoms than pH.
- Nodulation improved resistance to IDC in highly resistant and low resistant soybean genotypes.
- The developed assay is highly sensitive and can detect slight differences in IDC resistance.

## Introduction

Iron deficiency chlorosis (IDC) is an important nutrient deficiency in soybean (*Glycine max* (L.) Merr.), resulting in an estimated 260 million dollars in yield losses annually in the U.S. [20]. There are two major iron acquisition strategies known in plants. Strategy II plants (such as corn, oats, and other grasses) are able to take up Fe^3+^ under iron limiting conditions. This is accomplished by extrusion of siderophore compounds in the rhizosphere which bind Fe^3+^ and are then transported back inside the root zone where the iron can be converted to Fe^2+^ [14]. Strategy I plants, such as soybean, rely on the extrusion of protons to dissociate iron oxides through soil acidification, which then allows for Fe^3+^ to be reduced to Fe^2+^ in the rhizosphere and subsequently transported into the root. Several factors can limit the success of this strategy [14]. If the buffering capacity of the soil is too great, the plant may not be able to increase soil acidity enough to allow for iron to dissociate from iron oxide bonds [10, 11]. This strategy is also in direct competition with nitrogen acquisition. When nitrate is available in the soil, soybean often exacerbates iron stress by increasing the pH of the rhizosphere to scavenge nitrogen [2, 27]. Management of excess nitrogen in IDC prone areas is an important agronomic tool for limiting IDC symptoms [17].

IDC in soybean most frequently occurs in soils with high levels of calcium carbonates and pH greater than 7 [10], although the complete soil chemistry of iron deficiency in soybean is complex and can be exacerbated by other factors (i.e. nitrates). Genetic resistance to IDC remains the most effective method for managing IDC stress [9, 12]. Breeding for IDC resistance is challenging, relying on imperfect field observations in nurseries prone to IDC. Despite carefully selecting nurseries, symptoms may be absent in some years due to variation in soil moisture levels and climatic variables. IDC severity can present extreme spatial variation in nurseries, making standard comparisons between lines difficult [11, 28].

The lack of standard testing conditions in a controlled environment makes attempts at studying physiological differences between efficient and inefficient soybean genotypes especially challenging. Using soil from IDC nurseries in potted controlled environment experiments has been attempted, but results were not consistent between controlled environment type and environment from which soil was taken [3]. Fairbanks et al. [8] were able to achieve good IDC symptoms in controlled environments with soils from some IDC prone locations by ensuring proper water saturation of the soil. Soil collection, transport, and storage for experiments also brings into question the feasibility of this method. Many controlled IDC studies are done using hydroponic methods. Most often in these experiments, iron concentrations are limited in the nutrient solution [4] and pH is increased to induce IDC [7, 13, 19, 20]. Instead of making iron unavailable to plants, as occurs in the field, iron is limited in the hydroponic nutrient solution. The use of carbonates to limit iron and induce IDC symptoms in hydroponics has been conducted using solid NaHCO_3_ [5] or Mg(HCO_3_)_2_ [18] with carefully calibrated CO_2_ aeration. The use of a very fine liquid suspension source of CaCO_3_ could improve experimental results by allowing for better distribution and faster solubility of CaCO_3_. Coulombe et al. [5] also identified a possible independent effect of carbonates in the induction of IDC. The use of liquid suspended CaCO_3_ could allow for the independent study of the effects of pH and carbonates on soybean genotypes.

In controlled IDC hydroponic experiments, the effect of nodulation on IDC symptoms is largely ignored despite indications that nodulation can improve the response to iron stress in soybean [23]. A nodulation study of the cultivar T203, which has low resistance to IDC and does not respond to iron stress, showed that nodulated roots could produce an iron stress response and reduce chlorosis symptoms compared to non-nodulated plants [24]. Nitrogen fixation by *Bradyrhizobium japonicum* (henceforth referred to as *rhizobia)* bacteria is also a large iron sink for soybean. Leghemoglobin and nitrogenase, crucial enzymes involved in nitrogen fixation in the nodule, require iron for proper activity.

Soybean IDC resistance is a complex trait and there is a need to further dissect the factors and interactions that determine the plant’s symptoms and responses to this abiotic stress (Reviewed by [16]). Developing a method to represent field conditions more accurately will allow for a better understanding of the physiology of IDC in the future. The goal of this study was to develop a growth chamber assay to analyze the effects of genotype, nodulation, pH, and calcium carbonates on IDC severity using single potted plants as the experimental unit. The usefulness of such an assay would be determined by whether the assay can induce IDC symptoms in soybean and whether differences in IDC symptoms are found between efficient and inefficient soybean genotypes.

## Methods

### Plant materials

Two pairs of genotypes differing for resistance to IDC were selected for this study. To accommodate the large number of treatments required by the full factorial design of the experiment, only one pair of genotypes could be included in the growth chamber per experimental run and were thus analyzed separately. The first pair consisted of the cultivar ‘Corsoy 79’ (PI 518669), which has low resistance to IDC, and the cultivar ‘Dawson’ (PI 542403), which has high resistance to IDC [8]. ‘Corsoy 79’ and ‘Dawson’ are routinely used by the University of Minnesota soybean breeding program as low resistance and high resistance checks (respectively) of IDC in field nurseries, and are thus described as the “Check Genotypes Experiment” henceforth. The second pair consisted of near isogenic lines (NIL) that differ in resistance to IDC, developed from a cross between the genotypes ‘Fiskeby III’ (PI 438471) and ‘Mandarin (Ottawa)’ (PI 548379), as described by Merry et al. [15]. The specific pair used was from family 609 as described in Merry et al. [15]. The experiment involving these lines will henceforth be referred to as the “NIL Experiment”. The NIL genotypes are highly isogenic (F_11:12_) and differ at a ~ 137 kb region on Gm05 known to harbor a quantitative trait locus (QTL) controlling IDC resistance [15].

### Growth medium

Medium-coarse vermiculite (Sun Gro Horticulture Agawam, MA) was used as the growth medium in this study, as it is unreactive, devoid of nutrients which may complicate treatments, and maintains high moisture levels. Vermiculite was presoaked with tap water to saturation. For nodulated treatments, *rhizobia* (applied as TagTeam LCO granular inoculant (Novozymes A/S Bagsvaerd, Denmark)) was evenly mixed in vermiculite before water saturation to a ratio of 500 mL inoculant per 113 L (four cubic feet) of vermiculite. Some nodulation did occur in uninodulated pots (most likely from contaminant *rhizobia*), but was presumed inconsequential as the number of nodules formed was miniscule compared to inodulated plants and are thus described as unnodulated. Pots used in this experiment were 10.5 cm by 10.5 cm by 9 cm (length by width by depth, respectively) in dimension. Pots were filled with saturated vermiculite (~ 80 g dry weight vermiculite) and placed in individual plant saucers (15 cm diameter by 3.5 cm in deep) for treatment. Two to three seeds were planted per pot and were thinned to a single plant after emergence.

### Experimental design

**Fig. 1:**
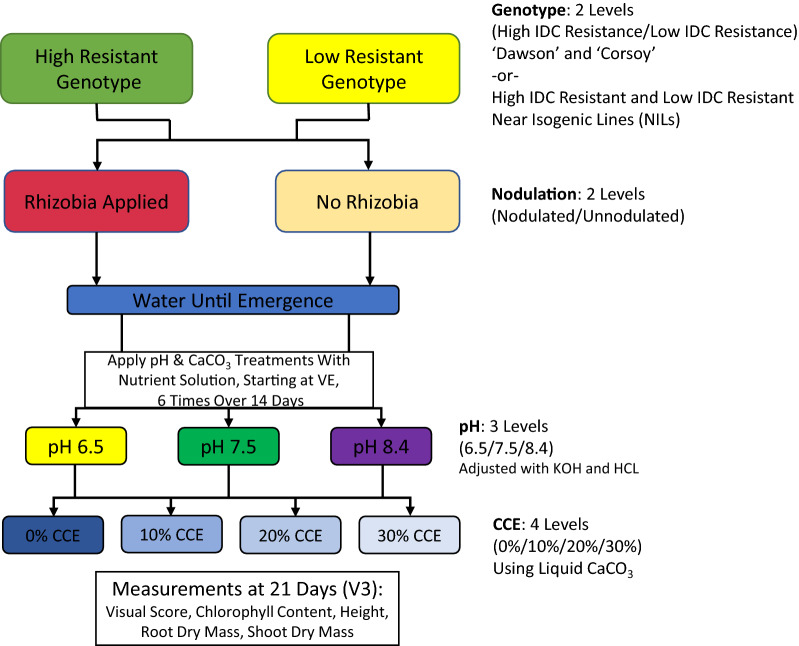
Overview of the full factorial experimental design and method. Each genotype was either inodulated with *rhizobia* at planting (thus resulting in nodulated plants) or uninodulated. One week after planting, a nutrient solution with pH and CCE treatment was applied to respective plants three times weekly for two weeks. After three weeks (21 days) plants were assessed for visual score, atLEAF value, height, root dry mass, and shoot dry mass

Seedlings were watered daily with tap water for the first week, after which nutrient solutions were used for treatment which were made using deionized water. A full factorial completely randomized design was used for treatments of individual plants, with 2 levels of genotype (efficient or inefficient), 2 levels of nodulation (inodulated with *rhizobia* and thus nodulated or uninodulated with *rhizobia* and thus unnodulated), 3 levels of pH (6.5, 7.5, and 8.4) and 4 levels of calcium carbonate, calculated as the calcium carbonate equivalency (CCE) (0%, 10%, 20%, 30%) (Fig. 1). A 5-12-26 (Nitrogen, Phosphorus and Potassium, respectively) hydroponic fertilizer containing 0.3% chelated iron (Jack’s Hydroponic 5-12-26 Part A fertilizer, J.R. Peters, Inc. Allentown, PA) was used as the nutrient source in all solutions at a rate of 0.96 g of fertilizer per L of water. The pH of treatment solutions was adjusted using KOH (5 mol) after the addition of any carbonates to solution. CAL-FLO liquid limestone (Burnett Lime Company, Inc. Campobello, SC) was used to adjust CCE, which was calculated as a dilution of CAL-FLO (64.17% CCE) to adjust the mass of the growing medium to the specified CCE. The range of CCE used in this study was selected to represent CCE values found in soils of the Midwestern U.S. [22]. Treatment solutions were applied 6 times over the course of 2 weeks (after the initial week of tap water) with complete replacement of solution at each application to ensure maintenance of pH throughout the experiment. Each saucer was filled with 300 mL of treatment solution to ensure equal amounts of solution were given to each plant and to allow for a free water surface in the saucer. Three single plant replications were used for each treatment combination per experiment. Because both experiments were repeated (for a total of two experimental runs), experimental run (henceforth described as “Run”) was included as a blocking factor.

### Growth chamber conditions

Growth chamber temperature averaged 25 °C for the duration of the experiment with 16 h of light and 8 h of dark per 24-h period. Light intensity during daylight hours averaged 32,000 Lux. Humidity was held constant at 70%. Growth chamber environmental conditions were monitored using a HOBO Data Logger (Onset Computer Corporation Bourne, MA) placed 10 cm from the chamber floor.

### Physiological measurements

Three weeks after planting, plants were removed from the growth chamber for analysis at the V4 growth stage. Plants were visually rated on a 1 to 9 scale with a rating of 1 being a completely healthy plant and a rating of 9 indicating plant death due to iron deficiency, as described by Merry et al. [15]. atLEAF value was used as a surrogate for chlorophyll content in this experiment and was measured using an atLeaf chlorophyll meter (FT Green LLC. Wilmington, DE). The atLeaf chlorophyll meter is an inexpensive tool with high correlations between atLEAF values and chlorophyll content [29]. atLEAF value of the 4th trifoliate was measured as the average of three readings, one from each leaflet of the trifoliate. Plant height was measured from the base of the plant to the topmost node. Roots were then cleaned of vermiculite, and the roots and shoots were separated and dried at 94 °C for 3–4 days until completely dry, at which time root dry mass (RDM) and shoot dry mass (SDM) were measured. All root nodules present were left intact for root dry weight measurements.

### Statistics

A full factorial ANOVA involving all factors and all combinations of their interactions and Run as a blocking factor was performed for each response variable using the aov function of the *stats* package (version 4.0.5) [21] in R (version 4.0.3). This was done separately for the Check Genotypes Experiment and the NIL Experiment. Significant differences between levels of a factor were determined using Tukey Honest Significant Differences (HSD) of the means.

## Results

### Genotype

Two separate genotype comparisons were performed in this study. The first, named the Check Genotypes Experiment, compared the cultivar ‘Corsoy 79’ (which has low resistance to IDC) and the cultivar ‘Dawson’ (which has high resistance to IDC). The second, named the NIL experiment, compared two isogenic lines that previously demonstrated differences in IDC resistance in field experiments, presumably due to variation at an IDC QTL on chromosome Gm05 [15]. These lines are described here as the ‘low resistant’ and ‘high resistant’ NILs, respectively, based on these field assessments. The known IDC severity difference between the two Check Genotype cultivars is relatively large, so we hypothesized that a good IDC phenotyping platform should differentiate their phenotypes. The IDC severity difference between the two NILs is relatively small, so we hypothesized that only a highly sensitive IDC phenotyping platform would differentiate their phenotypes.

The Check Genotype and NIL lines were each exposed to treatments that have been previously reported to impact IDC severity, including different combinations of pH level, CCE level, and nodulation. IDC symptom severity was assessed using several phenotypic measurements, including visual assessments of leaf coloration, atLEAF measurements of leaf chlorophyll content, plant height, RDM, and SDM. Reduced chlorophyll levels and interveinal yellowing of leaves, along with reduced height and biomass, were perceived as symptoms of increased IDC severity in these experiments.

Genotype was a significant source of variation for visual score (p < 0.001), height (p < 0.001), and SDM (p < 0.01) in the Check Genotypes Experiment (Table 1). ‘Corsoy 79’ had a higher average visual score compared to ‘Dawson’ (Table 2), indicating greater IDC symptoms, as expected. Furthermore, the ‘Dawson’ plants exhibited stronger growth and biomass, as the average height of ‘Dawson’ was significantly higher than ‘Corsoy 79’, and ‘Dawson’ also had a significantly higher average SDM than ‘Corsoy 79’ (Table 2). In the NIL Experiment, a significant difference due to genotype was identified in visual score (p < 0.01) and SDM (p < 0.001) (Table 1), with the highly resistant NIL having a better visual score and slightly greater shoot dry mass than the NIL with low IDC resistance (Table 3).

**Table 1:** Summary of significant effects in the “Check Genotypes Experiment” and the “NIL Experiment”

Source of variation	Visual score	atLEAF value	Height	Root dry mass	Shoot dry mass
Genotype	***CD/**NIL		***CD		**CD/**NIL
Inoculation	***CD/***NIL	***CD/***NIL	***CD/***NIL	***CD/***NIL	***CD/***NIL
pH	*CD	*CD	*CD	*CD	*CD
CCE	***CD/***NIL	***CD/***NIL	***CD/***NIL	***CD/***NIL	***CD/***NIL
Genotype × pH					
Genotype × CCE					
Genotype × Nodulation	*CD			**CD	*CD
Genotype × Nodulation × CCE	*NIL			*CD	**CD
Genotype × Nodulation × pH					
Genotype × CCE × pH					
CCE × pH					
Nodulation × pH					
Nodulation × CCE	**CD/*NIL	***CD	**CD		
Nodulation × CCE × pH					
Genotype × Nodulation × CCE × pH					
Run	***NIL	***CD/**NIL	***CD/***NIL	***CD/***NIL	***CD/**NIL

Significance code followed by ‘CD’ indicates significance in the Check Genotypes Experiment. Significance code followed by ‘NIL’ indicates significance in the NIL Experiment. Significance codes: (***) p < 0.001, (**) p < 0.01, (*) p < 0.05

**Table 2:** Main effects of the “Check Genotypes Experiment”

Source of variation	Level	Visual score (1–9 Scale)		atLEAF value		Height (cm)		Root dry mass (g)		Shoot dry mass (g)	
		Mean	SE	Mean	SE	Mean	SE	Mean	SE	Mean	SE
Genotype	'Corsoy 79'	4.5^a^	0.16	32.3^n.s^	0.50	11.1^a^	0.23	0.62^n.s^	0.02	1.1^a^	0.04
	'Dawson'	3.8^b^	0.13	31.9^n.s^	0.41	12.0^b^	0.22	0.65^n.s^	0.02	1.2^b^	0.04
Nodulation status	Nodulated	3.5^a^	0.12	33.7^a^	0.37	12.4^a^	0.24	0.69^a^	0.02	1.3^a^	0.04
	Unnodulated	4.8^b^	0.16	30.8^b^	0.50	10.8^b^	0.20	0.58^b^	0.02	1.0^b^	0.04
pH	6.5	3.9^a^	0.19	32.4^ab^	0.59	11.7^ab^	0.30	0.66^a^	0.03	1.2^a^	0.06
	7.5	4.1^ab^	0.18	33.1^a^	0.55	11.8^a^	0.28	0.65^ab^	0.03	1.2^a^	0.05
	8.5	4.5^b^	0.18	31.4^b^	0.53	11.2^b^	0.26	0.59^b^	0.03	1.1^b^	0.05
CCE%	0%	2.8^a^	0.14	36.7^a^	0.47	12.8^a^	0.31	0.71^a^	0.04	1.6^a^	0.07
	10%	4.1^b^	0.19	32.0^b^	0.51	11.5^b^	0.34	0.66^ab^	0.03	1.1^b^	0.05
	20%	4.5^b^	0.20	30.6^bc^	0.60	11.2^b^	0.29	0.62^bc^	0.03	1.0^bc^	0.05
	30%	5.3^c^	0.20	29.9^c^	0.67	10.9^b^	0.31	0.55^c^	0.03	0.9^c^	0.04

Main Effects of the Check Genotypes Experiment and means and standard error (S.E.) for each measured trait pooling both runs (n = 6). Significant differences between means within each source of variation were determined using Tukey’s HSD test and are indicated with differing letter superscripts

**Table 3:** Main Effects of the NIL genotypes experiment

Source of variation	Level	Visual score (1–9 Scale)		atLEAF value		Height (cm)		Root dry mass (g)		Shoot dry mass (g)	
		Mean	SE	Mean	SE	Mean	SE	Mean	SE	Mean	SE
Genotype	'Susceptible NIL'	4.1^a^	0.14	26.7^n.s^	0.53	10.3^n.s^	0.18	1.4^n.s^	0.09	1.5^a^	0.06
	'Tolerant NIL'	3.8^b^	0.15	26.4^n.s^	0.53	10.6^n.s^	0.18	1.3^n.s^	0.09	1.6^b^	0.07
Nodulation status	Nodulated	3.0^a^	0.11	30.1^a^	0.31	11.3^a^	0.19	1.6^a^	0.1	1.7^a^	0.07
	Unnodulated	4.9^b^	0.14	22.6^b^	0.5	9.6^b^	0.14	1.2^b^	0.08	1.3^b^	0.06
pH	6.5	3.8^n.s^	0.17	26.1^n.s^	0.66	10.7^n.s^	0.22	1.5^n.s^	0.12	1.5^n.s^	0.07
	7.5	4.0^n.s^	0.17	26.5^n.s^	0.6	10.4^n.s^	0.21	1.3^n.s^	0.09	1.5^n.s^	0.08
	8.5	4.0^n.s^	0.2	27.1^n.s^	0.69	10.3^n.s^	0.24	1.4^n.s^	0.12	1.5^n.s^	0.09
CCE%	0%	2.0^a^	0.14	30.5^a^	0.69	11.9^a^	0.27	2.0^a^	0.19	2.7^a^	0.07
	10%	4.4^b^	0.15	25.7^b^	0.68	10.2^b^	0.25	1.2^b^	0.09	1.2^b^	0.04
	20%	4.8^b^	0.17	24.9^b^	0.67	9.9^b^	0.21	1.2^b^	0.09	1.1^b^	0.03
	30%	4.7^b^	0.17	25.1^b^	0.77	9.9^b^	0.22	1.2^b^	0.09	1.1^b^	0.04

Main Effects of the NIL Genotypes Experiment and means and standard error (S.E.) for each measured trait pooling both runs (n = 6). Significant differences between means within each source of variation calculated with Tukey’s HSD test and are indicated with differing letter superscripts

### Calcium carbonate

Calcium carbonate level was a significant source of variation for all phenotypes measured (p < 0.001 for visual score, atLEAF value, height, RDM, and SDM) in both the Check Genotypes Experiment and NIL Experiment (Table 1). In the Check Genotypes Experiment, plants with 0% CCE had the lowest average visual score, indicating the least severe IDC symptoms. Plants with 10% or 20% CCE were not statistically significant from one another but had a significantly higher average visual score than plants with 0% CCE, as well as a significantly lower average visual score than plants with 30% CCE. Plants with 30% CCE had a significantly higher visual score than all other levels of CCE (Table 2), indicating the most severe IDC symptoms. In a similar pattern, atLEAF values significantly decreased with increasing CCE levels (Table 2), indicating reduced chlorophyll content in the higher CCE treatments**.** Furthermore, plants given 0% CCE were significantly taller than plants given 10%, 20%, and 30% CCE of calcium carbonate. However, the heights of plants given calcium carbonate at 10%, 20%, and 30% CCE were not significantly different from each other (Table 2). Root dry mass and SDM decreased with increasing CCE (Table 2).

For the NIL Experiment, plants with 0% CCE had the lowest average visual score, again indicating the least severe IDC symptoms. Plants with 10% CCE, 20% CCE, and 30% CCE had visual scores significantly higher than plants with 0% CCE, but not significantly different from each other (Table 3). Similar patterns were found for atLEAF value, height, RDM, and SDM, in which plant averages at 0% CCE were significantly better than plants given 10%, 20%, or 30% CCE treatments (Table 3).

### Effect of pH

Significant differences between levels of pH were found for all response variables in the Check Genotypes Experiment (p < 0.05 for visual score, atLEAF value, plant height, RDM, and SDM) (Table 1). pH was not, however, a significant source of variation in the NIL Experiment (Table 1). For visual score in the Check Genotypes Experiment, plants given nutrient solution with a pH adjusted to 6.5 had an average visual score similar to plants treated with a pH of 7.5. Plants treated with a pH of 8.4 had similar visual scores as plants treated with a pH of 7.5, but had significantly higher visual scores than plants given the 6.5 pH treatment (Table 2). For atLEAF value, plants treated with a pH of 6.5 were not significantly different than plants treated with a pH of 7.5 or 8.4. However, plants treated with a pH of 7.5 had significantly higher atLEAF value than plants treated with a pH of 8.4 (Table 2). Correspondingly, the height of plants treated with a pH of 6.5 and 7.5 were similar, but the pH 7.5 treatment resulted in significantly taller plants than those treated with a pH of 8.4 (Table 2). RDM and SDM followed a more expected pattern, with increased pH decreasing mass in both traits (Table 2). These patterns indicate that changes in pH have a subtle effect on the chlorosis phenotype in the Check Genotypes Experiment which is not always consistent between the different traits that were measured.

### Nodulation status

Nodulation status in this experiment is referred to as inodulated with *rhizobia* and thus nodulated or uninodulated with *rhizobia* and thus unnodulated. In the Check Genotypes Experiment, nodulation status was a significant source of variation for visual score (p < 0.001), atLEAF value (p < 0.001), height (p < 0.001), RDM (p < 0.001), and SDM (p < 0.001) (Table 1). Nodulated plants had a lower visual score on average compared to that of non-nodulated plants (Table 2), indicating increased plant health. Nodulated plants also had higher average atLEAF values compared to non-nodulated plants, were significantly taller, and had higher RDM and SDM (Table 2). Significant differences for visual score (p < 0.001), atLEAF value (p < 0.001), height (p < 0.001), RDM (p < 0.001), and SDM (p < 0.001) were also observed between nodulated and non-nodulated plants in the NIL Experiment (Table 1), following similar trends as those in the Check Genotypes Experiment (Table 3).

### Genotype × Nodulation interaction

**Fig. 2:**
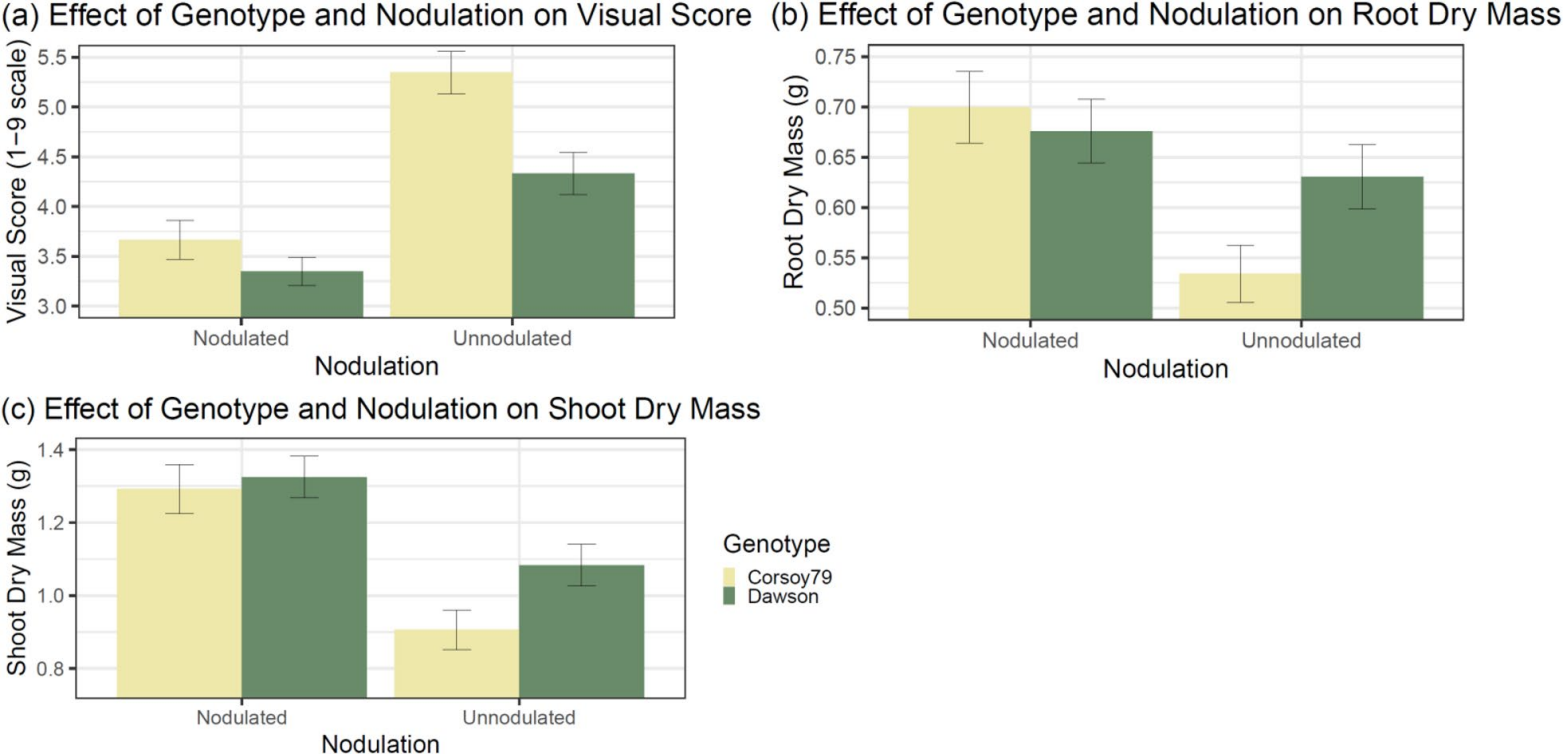
Effect of Genotype and Nodulation on Visual Score (**a**), Root Dry Mass (**b**), and Shoot Dry Mass (**c**) in the Check Genotypes Experiment. Error bars represent ± 1 SE, n = 72

The Genotype × Nodulation interaction was significant for visual score (p < 0.05), RDM (p < 0.01), and SDM (p < 0.05) for the Check Genotypes Experiment (Table 1). For the Check Genotypes Experiment, nodulated genotypes had a similar average visual score. The genotype ‘Corsoy 79’ had a higher average visual score compared to the average visual score of genotype ‘Dawson’ when plants were unnodulated. Unnodulated plant visual scores were significantly higher than nodulated plant visual scores for both genotypes (Fig. 2a), indicating increased plant health with nodulation. For RDM, ‘Dawson’ when both nodulated and unnodulated had similar root mass to nodulated ‘Corsoy 79’. Unnodulated ‘Corsoy 79’ had a significantly lower root mass than nodulated ‘Corsoy 79’ and ‘Dawson’ when nodulated and unnodulated (Fig. 2b), potentially revealing reduced nutrient scavenging abilities in ‘Corsoy 79’ compared to ‘Dawson’. In the Genotype × Inoculation interaction for SDM, ‘Corsoy 79’ and ‘Dawson’ had significantly higher shoot dry mass when nodulated but were not different from each other. Unnodulated ‘Dawson’ was significantly lower in SDM than either nodulated genotype. Unnodulated ‘Corsoy 79’ had the lowest SDM, which was different from all other treatments in the interaction (Fig. 2c).

### Nodulation × CCE interaction

**Fig. 3:**
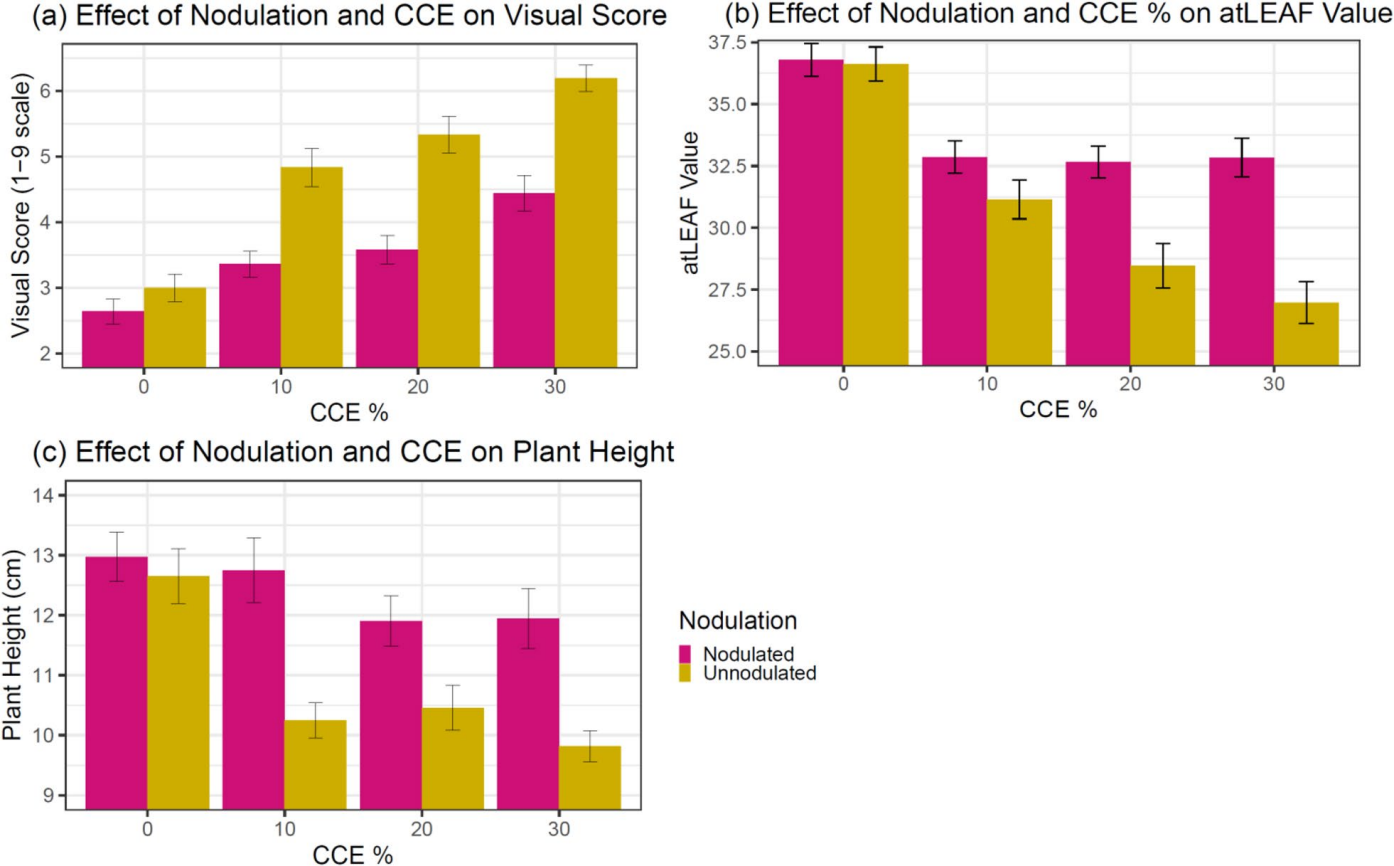
Effect of Nodulation and Calcium Carbonate Equivalent on Visual Score (**a**), atLEAF Value (**b**), and Plant Height (**c**) in the Check Genotypes Experiment. Error bars represent ± 1 SE, n = 38

**Fig. 4:**
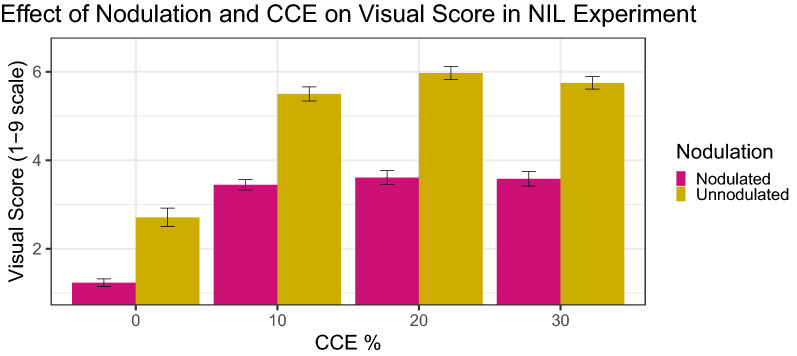
Effect of Nodulation and Calcium Carbonate Equivalent on Visual Score in the NIL Genotypes Experiment. Error bars represent ± 1 SE, n = 38

The Nodulation × CCE% interaction was significant for visual score in both the Check Genotypes Experiment and NIL Experiment (p < 0.01 and p < 0.05, respectively) (Table 1). For the Check Genotypes Experiment, nodulated plants with 0%, 10%, and 20% CCE as well as unnodulated plants with 0% CCE had similar average visual scores. Nodulated plants with 20% CCE had a similar visual score to nodulated plants with 30% CCE. Unnodulated plants with 10% or 20% CCE had average visual scores similar to nodulated plants with 30% CCE, showing that nodulated plants can withstand higher carbonate pressure. Unnodulated plants with 30% CCE had the highest average visual score but were not significantly different from unnodulated plants with 20% CCE (Fig. 3a). In the NIL Experiment, nodulated and unnodulated plants at 0% CCE had significantly lower visual scores than all other levels (although not significantly different from each other at 0% CCE) (Fig. 4). It is logical that plants with no carbonates would be healthier compared to plants with adjusted CCE% treatments. Nodulated plants at 10%, 20%, and 30% CCE had similar visual scores to each other, which were significantly lower than unnodulated plants at 10%, 20%, and 30% CCE. Unnodulated plants at 10%, 20%, and 30% CCE had similar visual scores, which were significantly higher than all other levels (Fig. 4).

atLEAF value (p < 0.001) and height (p < 0.01) also had a significant Nodulation × CCE% interaction in the Check Genotypes Experiment (Table 1). In general, the atLEAF value continued to decrease for unnodulated plants as CCE% increased, while atLEAF value decreased from 0 to 10% CCE but remained stable at higher CCE% for nodulated plants (Fig. 3b), exemplifying the benefits of inoculation with *rhizobia*. Nodulated plants at all CCE% levels and unnodulated plants at 0% CCE were similar in height, and significantly taller than unnodulated plants at 10%, 20%, and 30% CCE (Fig. 3c).

### Genotype × Nodulation × CCE% interaction

**Fig. 5:**
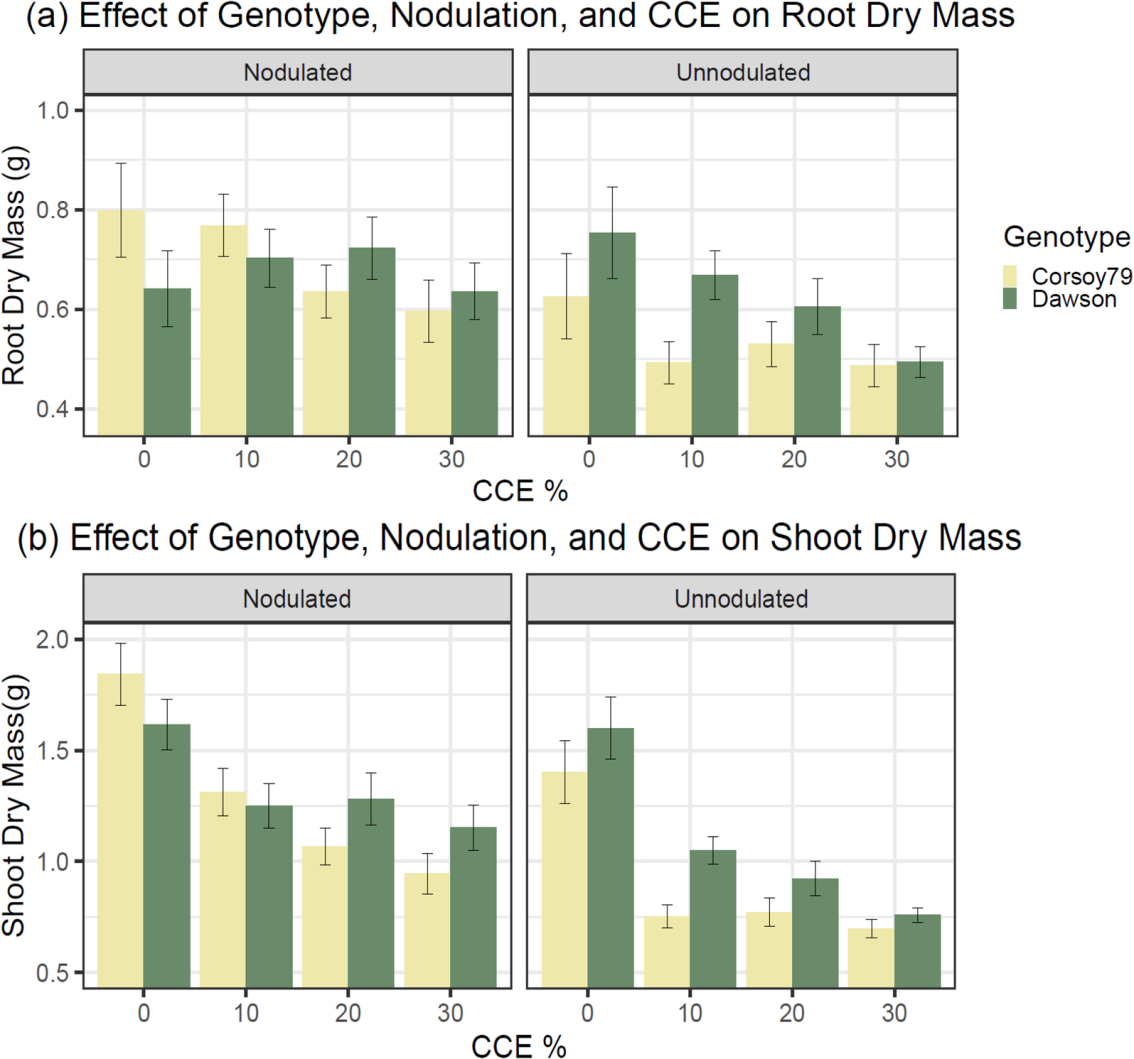
Effect of Genotype, Nodulation, and Calcium Carbonate Equivalent on **a** Root Dry Mass and **b** Shoot Dry Mass in the Check Genotypes Experiment. Error bars represent ± 1 SE, n = 20

The Genotype × Nodulation × CCE% three-way interaction was significant for RDM (p < 0.05, Fig. 5a) and SDM (p < 0.01, Fig. 5b) in the Check Genotypes Experiment (Table 1). In the Check Genotypes Experiment, the unnodulated genotype ‘Corsoy 79’ had an immediate reduction in RDM and SDM at 10% CCE, which was maintained as CCE levels increased. Nodulated ‘Corsoy 79’ more gradually decreased RDM and SDM with increasing CCE levels. The genotype ‘Dawson’ maintained higher RDM and SDM than ‘Corsoy 79’ when unnodulated at all levels of CCE, with a similar gradual reduction found in nodulated ‘Corsoy 79’ with increasing CCE. Nodulated ‘Dawson’ maintained RDM at all levels of CCE and maintained SDM at higher CCE levels after an initial decrease at 10% CCE.

**Fig. 6:**
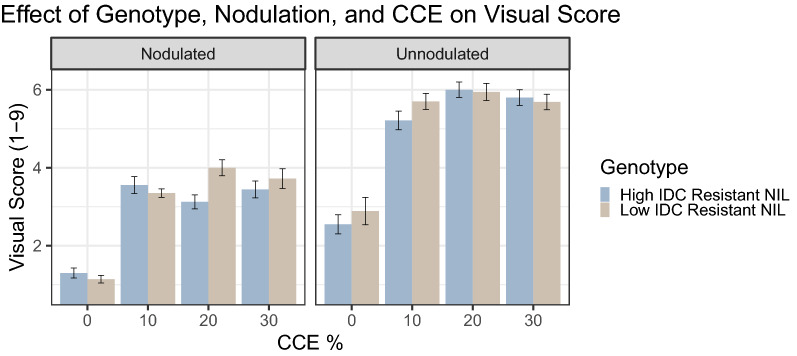
Effect of Genotype, Nodulation, and Calcium Carbonate Equivalent on Visual Score in the NIL Genotypes Experiment. Error bars represent ± 1 SE, n = 20

In the NIL experiment, the Genotype × Nodulation × CCE% three-way interaction was significant for visual score (p < 0.05, Table 1, Fig. 6). Unnodulated plants had higher visual scores (indicating increased symptoms) at all levels of CCE compared to nodulated plants of the same genotype. While the efficient and inefficient NIL had similar visual scores at most levels of Nodulation × CCE, the efficient NIL genotype had slightly lower visual scores when nodulated and at 20% CCE, and when unnodulated and at 10% CCE.

## Discussion

The main objective of this study was to develop a controlled-environment assay to elucidate the effects of genotype, nodulation, pH, and calcium carbonates on IDC. Ideally, a single combination of specific levels of the previously mentioned stress factors could be used to maximize differences in IDC symptom differences between iron-efficient and inefficient genotypes. This would make it possible to increase the number of replicates and develop a simple “control” and “treatment” experimental design to assess IDC resistance among genotypes. The significant three-way interactions between genotype, nodulation, and CCE% are critical as they indicate potential differentiation between efficient and inefficient genotypes in IDC resistance using this assay. The two genotype comparisons in this study (Check Genotypes vs. NIL Genotypes) displayed their greatest pairwise differences when the CCE was adjusted to 20% (regardless of pH). However, optimal nodulation status and response variable behavior were not the same between genotype comparisons. For example, the greatest difference between Check Genotypes was found for RDM (Fig. 5a) and SDM (Fig. 5b) when the plants were unnodulated and the CCE adjusted to 10% or 20%. However, only visual score had a significant interaction with genotype in the NIL comparison. The greatest differences between NIL genotypes occurred in the visual score when nodulated at 20% CCE (Fig. 6). These findings indicate that optimal treatment conditions and the best response variable to measure for IDC severity can vary for different genotype comparisons when using this method. The optimal experimental parameters are not universal for all genotype comparisons. In our experiments, it is possible that the differential genetics of the Check Genotypes comparison (polygenic) and the NIL Genotypes comparison (single locus) may have influenced the optimal experimental parameters to elicit differences in IDC phenotypes, due to the genes and biological processes influencing the respective comparisons.

As expected, increased pH worsened IDC symptoms in the Check Genotypes for visual score, atLEAF value, height, RDM, and SDM (Table 2) but was surprisingly not a significant source of variation in the NIL experiment (Table 3). Varying effects of pH between the Check Genotypes and the NIL Genotypes suggests the existence of multiple types of resistance to iron deficiency. While the check genotypes are affected by both pH and carbonate sources of iron stress, the NIL genotypes overcome iron stress due to pH but not increased carbonates. Iron reduction in the rhizosphere is the primary mechanism of iron acquisition in Strategy I plants [14]. It is possible that the NIL genotypes are better able to reduce Fe^3+^ to Fe^2+^ in the rhizosphere than the check genotypes, and are thus less affected by higher soil pH per se.

It was expected that a significant interaction would be found between pH and CCE%, however no significance was found for this interaction in either experiment for any measured traits. In fields with calcareous soils, high soil pH often results in the release of bicarbonates [10, 11]. Bicarbonate release potentially has a greater effect on IDC severity than pH per se, but in field conditions this is nearly impossible to decouple. Further dissection of the relationship between carbonates and pH using this assay could help to improve our understanding of IDC in soybean.

Iron deficiency symptoms for all traits occurred with the treatment of calcium carbonates in both the Check Genotypes Experiment (Table 2) and the NIL Experiment (Table 3). While symptoms showed increased severity for the check genotypes with increases in CCE%, no significant differences were found in any traits in the NIL Experiment among CCE levels higher than 10%. It is possible that the NIL genotypes were better able to maintain iron homeostasis with increasing carbonate levels. Alternatively, they may be immediately overwhelmed with the addition of 10% CCE and maintain their physiological limit at higher levels of CCE. This could be discerned by further increasing the percent CCE to see if symptoms become more severe or are still maintained, although in field conditions CCE rarely exceeds 30%.

As an often-overlooked factor in IDC studies, a discussion on symbiosis is warranted here. Inoculation with *rhizobia* significantly improved all measured traits in the Check Genotypes Experiment (Table 2) and NIL Genotypes Experiment (Table 3). This is unsurprising when considered alone, as nodulation and nitrogen fixation by soybean has been well documented to improve yield more than nitrogen fertilizer [1, 25, 26].

Nodulation was the only factor that was a component in all significant interactions in this study (Table 1). Of particular interest and importance for IDC are the significant two-way interaction of nodulation with CCE and the significant three-way interaction between nodulation, genotype and CCE. For all significant interactions of nodulation with CCE, the addition of calcium carbonates exacerbated IDC symptoms. However, unnodulated plants had more severe IDC symptoms than nodulated plants as CCE% increased, similar to findings by Soerensen et al. [23]. It was assumed that nitrogen deficiency was not the cause of this observation because differences between efficient and inefficient genotypes did not occur until CCE% was increased. With advances in molecular techniques, a better dissection of the relationship between nodulation and IDC stress may be timely.

Investigating genotypes with small differences in resistance to IDC in a controlled environment is novel. Previous controlled environment IDC studies have focused on genotypes with sizeable differences in IDC resistance [5, 7, 13, 19, 20]. While the NILs used in this study did not have a significant interaction with genotype and pH or CCE% in any of the individual symptoms of IDC, the interaction of genotype × inoculation × CCE level was significant with visual score. While the efficient and inefficient NILs had similar visual scores at many treatment levels, the differentiation found when nodulated and at 20% CCE and when unnodulated and at 10% CCE followed the expected pattern of the inefficient genotype having a higher visual score indicating less resistance to IDC. The one-point difference in visual score found when nodulated and at 20% CCE in this study is close to the 1.5-point difference for the NIL pair found by Merry et al. [15] in field conditions.

The significant difference in SDM between the NIL genotypes is intriguing. The NIL pair used in this study is highly isogenic (F_11_-derived) and developed to express differences in resistance to IDC at a single genetic locus, thus any physiological differences between the NIL genotypes should be considered as a potential mechanism for IDC resistance. Because most studies focus on resultant phenotypes after IDC stress, investigating differences in pre-stress plant physiology to identify potential iron efficiency traits may add novel information to this area of study.

It is important to note that the method described here is practical for studying IDC response differences among a relatively small number of genotypes. However, it is unlikely that further refinements could be made to successfully score a large number of genotypes for IDC resistance, as is routinely done in a breeding program, using this method. There are several reasons for this conclusion. First, the number of replicates required to accurately rate IDC severity makes space requirements limiting as the number of genotypes increases. Second, the time required to apply nutrient treatments and measure traits on a large number of genotypes in controlled conditions is far more expensive than visually rating plants in an IDC field nursery or taking automated measurements with drone images from field data [6]. Lastly, a “universal” optimal treatment for a large set of genotypes would not give accurate IDC resistance ratings using the approach described here. This is indicated by the different optimal combination of inoculation and CCE that were identified for the Check Genotypes and NIL Genotypes for inducing IDC symptoms. While these limitations make examining many genotypes impractical, this method can still be utilized for advancing our understanding of the genetics and physiology of IDC through comparisons of efficient and inefficient genotypes, as well as decoupling the effects of pH and CCE on IDC.

## Conclusion

IDC symptoms were successfully induced in the Check Genotypes Experiment as well as the NIL Experiment, indicating the success of using a liquid suspension of CaCO_3_ and this assay for inducing IDC in controlled environments. Sensitivity of the assay was highest when comparing ‘Corsoy 79’ and ‘Dawson’, which have large differences in IDC resistance. The greatest significant differences between ‘Corsoy 79’ and ‘Dawson’ were shown in the RDM and SDM phenotypes when plants were unnodulated and treated with 10% CCE. Meanwhile, using this approach to identify IDC differences among genotypes with small resistance differences may require more replication and optimization of the experimental treatments such as alternative levels of CCE. Interestingly, CCE and pH impacted IDC severity independently, as indicated by a lack of a significant interaction between the two factors, and pH was not a significant factor in the NIL Experiment for any measured trait. Nodulation reduced IDC symptoms in both pairs of genotypes and is valuable to include as a factor when examining the physiological response of soybean to iron stress.

## Data Availability

The data generated by this study is available upon the approval of Aaron Lorenz and Bob Stupar from the University of Minnesota Department of Agronomy and Plant Genetics.

## Abbreviations

CCE: Calcium carbonate equivalency
IDC: Iron deficiency chlorosis
NIL: Near isogenic line
RDM: Root dry mass
SDM: Shoot dry mass
